# Clinical implications of rapid ePlex® Respiratory Pathogen Panel testing compared to laboratory-developed real-time PCR

**DOI:** 10.1007/s10096-017-3151-0

**Published:** 2017-12-08

**Authors:** Anneloes L. van Rijn, Roel H. T. Nijhuis, Vincent Bekker, Geert H. Groeneveld, Els Wessels, Mariet C. W. Feltkamp, Eric C. J. Claas

**Affiliations:** 1 Department of Medical Microbiology, Leiden University Medical Center, P.O. Box 9600, 2300 RC Leiden, The Netherlands; 2 Department of Pediatrics, Leiden University Medical Center, Leiden, The Netherlands; 3 Department of Internal Medicine, Leiden University Medical Center, Leiden, The Netherlands

## Abstract

Rapid diagnosis of respiratory infections is of great importance for adequate isolation and treatment. Due to the batch-wise testing, laboratory-developed real-time polymerase chain reaction (PCR) assays (LDT) often result in a time to result of one day. Here, LDT was compared with rapid ePlex® Respiratory Pathogen (RP) Panel testing of GenMark Diagnostics (Carlsbad, CA, USA) with regard to time to result, installed isolation precautions, and antibacterial/antiviral treatment. Between January and March 2017, 68 specimens of 64 patients suspected of an acute respiratory infection were tested with LDT and the ePlex® RP panel. The time to result was calculated as the time between sample reception and result reporting. Information regarding isolation and antibacterial/antiviral treatment was obtained from the patient records. Thirty specimens tested LDT positive (47%) and 29 ePlex® RP panel positive (45%). The median time to result was 27.1 h (range 6.5–96.6) for LDT versus 3.4 h (range 1.5–23.6) for the RP panel, *p*-value < 0.001. In 14 out of 30 patients, isolation was discontinued based on the ePlex® RP panel results, saving 21 isolation days. ePlex® RP panel test results were available approximately one day ahead of the LDT results in the 19 patients receiving antiviral/antibacterial treatment. In addition, two bacterial pathogens, not requested by the physician, were detected using the RP panel. Analysis of respiratory infections with the ePlex® RP panel resulted in a significant decrease in time to result, enabling a reduction in isolation days in half of the patients. Furthermore, syndromic RP panel testing increased the identification of causative pathogens.

## Introduction

Respiratory tract infections are a leading cause of hospital admission, morbidity, and mortality [1–4]. At presentation, etiological agents of the respiratory tract infection cannot be identified solely based on clinical signs and symptoms. Therefore, and awaiting microbiological confirmation, empirical antibiotic and antiviral treatment is initiated based on severity score and the influenza season [5]. Since only a minority of the infections is being caused by bacteria, this empiric antibiotic treatment approach is redundant and can lead to an increase in antibiotic resistance. Moreover, empiric isolation precautions are installed to protect other patients and healthcare workers from a possible (viral) infection. Altogether, there is a need for rapid identification or exclusion of a viral respiratory tract infection to reduce inappropriate (unnecessary) hospital hygienic interventions and focus (shorten) antibacterial/antiviral treatment.

Currently, the diagnosis of respiratory infections is usually based on (a combination of) molecular amplification methods and bacterial culture. In our laboratory, laboratory-developed real-time polymerase chain reaction (PCR) multiplex assays (LDT) are used that show excellent sensitivity and specificity. However, this approach is limited by the number of targets per multiplex reaction and the need for batch-wise testing. The assays are performed once daily, with a time to result of approximately 20 h.

Recently, the Respiratory Pathogen (RP) Panel of GenMark Diagnostics (Carlsbad, CA, USA) has become available for the detection of an extensive panel of respiratory pathogens (21 respiratory viruses, three bacterial species; see the Methods section) using eSensor technology [6]. This test is a cartridge-based molecular assay to be used on the ePlex® platform with a time to result of approximately 90 min that showed a concordance of > 97% compared to LDT [7]. Hypothetically, ePlex® RP panel testing represents a considerable reduction in time to diagnosis, as compared to LDT, which could have significant clinical benefits. In this paper, a pilot study is reported that analyzed the implications of using the ePlex® RP panel for the detection of respiratory infections compared to LDT regarding time to result, isolation precautions, and antibacterial/antiviral therapy.

## Methods

### Inclusion of patients

This prospective, single-center study in the Leiden University Medical Center (LUMC) included patients from January to March 2017. Patients with symptoms of an acute respiratory infection were included upon request of the physician of the acute ward, intensive care unit, and pediatric department. Specimens included were obtained during weekdays and tested with both the RP panel and LDT after consulting the microbiologist. Information regarding baseline characteristics, infection parameters, admittance, isolation, and treatment was obtained from the electronic patient records. Additional information about cultures was retrieved from the laboratory information system (GLIMS, MIPS, Belgium). The medical ethics review committee of the LUMC approved the study.

#### Primary outcome measure

The primary endpoint of this study was the time to result of the ePlex® RP panel compared to LDT.

#### Secondary outcome measures

The ePlex® RP panel was offered as a pilot to elevate the pressure on droplet isolation rooms; thus, isolation was discontinued based on the ePlex® RP panel results. Due to the pilot nature of this study, antibacterial and antiviral treatment were not adjusted based on the ePlex® RP panel results; therefore, only the theoretical time reduction in treatment was calculated using the time to results of the ePlex® RP panel and LDT. Secondary outcome measures were the reduction of isolation days based on the ePlex® RP panel ahead of LDT results, the theoretical reduction in hours in oseltamivir and atypical pneumonias treatment calculated with the time to results of LDT and the ePlex® RP panel, and possible additional diagnosis found with the ePlex® RP panel.

### Laboratory-developed testing (LDT)

LDT viral testing and testing for *Mycoplasma pneumoniae*, *Chlamydia pneumoniae*, and *Chlamydia psittaci* was performed the same day on all samples that arrived at the laboratory before 8:15 A.M. Samples arriving at the laboratory before 3:30 P.M. were tested for *Legionella pneumophila* and *Bordetella parapertussis* the following day. These assays were performed daily from Monday till Friday and on request on weekend days. The viral respiratory panel of LDT consists of adenovirus, bocavirus, coronavirus 229E, coronavirus HKU1, coronavirus NL63, coronavirus OC43, influenza A, influenza B, human metapneumovirus, parainfluenza 1–4 (differentiation with differently labeled probes), respiratory syncytial virus, and rhinovirus. In addition, testing for bacterial pathogens could be requested: *Legionella* species, *Legionella pneumophila*, *Mycoplasma pneumoniae*, *Chlamydia pneumoniae*, *Chlamydia psittaci*, *Bordetella pertussis*, and *Bordetella parapertussis*.

All sputa samples were 1:5 diluted in phosphate-buffered saline (PBS) and homogenized by bead-beating prior to extraction. Then, 200 μL of each respiratory sample was used to extract 100 μL total nucleic acids using the Total Nucleic Acid Extraction kit on the MagNA Pure LC system (Roche Diagnostics). Nucleic acid amplification and detection by real-time PCR was performed on a Bio-Rad CFX96 thermocycler, using primers, probes, and conditions as described previously [8–10]. For the detection of *Mycoplasma pneumoniae*, *Chlamydia pneumoniae*, and *Chlamydia psittaci* the b-CAP assay (Biolegio, Nijmegen, the Netherlands) developed for the BD-MAX system was used by testing 200 μL of each respiratory sample according to the manufacturer’s instructions [11]. LDT results were reported in the electronic patient record. The time to result for the LDT was calculated as the time from receipt of the sample in the laboratory to the time results were available in the electronic patient record.

### ePlex® RP panel

Specimens for diagnosis using the CE-IVD cleared RP panel were accepted on weekdays between 8:15 A.M. and 3:00 P.M. and tested during the day, as soon as possible. The ePlex® RP panel was not offered during the weekend, while treatment was not adjusted based on the results. The RP panel as used in the study was able to detect adenovirus, bocavirus, coronavirus 229E, coronavirus HKU1, coronavirus NL63, coronavirus OC43, influenza A H1, influenza A 2009 H1N1, influenza A H3, influenza B, metapneumovirus, Middle East respiratory syndrome coronavirus, parainfluenza 1–4, respiratory syncytial virus A and B, rhinovirus/enterovirus, *Bordetella pertussis*, *Legionella pneumophila*, and *Mycoplasma pneumoniae*. As with LDT, sputa samples were diluted in an 1:5 dilution using PBS. According to the manufacturer’s instructions, 200 μL of the respiratory sample was pipetted in a buffer tube and, after vortexing, transferred to the ePlex cartridge and, subsequently, to the ePlex tower. If the test gave an invalid result, the run was repeated. Results were reported by telephone to the requesting physician, since the results were not reported in the electronic patient record. The time to result was calculated as the time from receipt of the sample to the time results were reported by telephone.

### Statistics used for comparison

The time to result was compared with the Wilcoxon signed-rank test, using IBM SPSS Statistics version 23 software for Windows. A *p*-value < 0.05 was considered statistically significant.

## Results

### LDT and ePlex® RP panel results

Between January and March 2017, 64 patients were included with symptoms of acute respiratory infection, whose characteristics are summarized in Table 1. A total of 68 samples were tested, comprising 40 throat swabs, 13 sputum samples, 11 nasal lavages, and four nasopharyngeal swabs. Thirty-four tested positive for a respiratory pathogen in one or both assays. Six samples failed in the ePlex® RP panel, of which two gave a valid result upon retesting. The other four were not retested, two because of insufficient remaining sample volume. The failed samples, if not retested, were excluded from further analysis, leaving 64 samples of 61 patients for further analysis. None of the samples failed in the LDT.

**Table 1:** Patient characteristics

	Patients, *n* = 64	Range or %
Demographics		
Age, median years (range)	60	0–93
Male sex (%)	33	52
Clinical features		
Diagnosis		
Pneumonia (%)	25	39
COPD/asthma exacerbation (%)	7	11
RTI other than pneumonia (%)	12	19
Other diagnosis (%)	20	31
Leukocytes, median ×10^9^/L (range)	11.4	0.44–49.16
C-reactive protein level, median mg/L (range)	62	2–360
Cough (%)	49	77
Sputum (%)	26	41
Previous antibiotic treatment (%)	20	31
Duration of symptoms, median days (range)	2	1–21
Comorbidity		
COPD/asthma (%)	17	27
Diabetes (%)	7	11
Malignancy (%)	6	9
Transplantation (%)	12	19
Autoimmune disease (%)	8	13
Admission ward		
Acute ward	32	50
Intensive care (including children)	8	13
Pediatric department	8	13
Other departments	15	23
Not admitted	1	1

*COPD* Chronic obstructive pulmonary disease; *RTI* respiratory tract infection

Of the 64 samples, 31 tested positive for a total of 37 pathogens with LDT or the ePlex® RP panel (Table 2). Using LDT, 30 tested positive and 34 negative, whereas this was 29 and 35 using the ePlex® RP panel. As shown in Table 3, a discordant result was found in five samples.

**Table 2:** Respiratory pathogens found in clinical samples with the laboratory-developed real-time polymerase chain reaction (PCR) assay (LDT) or the ePlex® Respiratory Pathogen (RP) Panel

Pathogens	LDT	ePlex® RP panel
Coronavirus 229E	2	2
Coronavirus HKU1	1	1
Human bocavirus	1	1
Human metapneumovirus	5	4
Influenza A	10	9 (all H3)
Influenza B	1	1
Parainfluenza virus type 3	1	1
Respiratory syncytial virus	4	0
Respiratory syncytial virus type A		2
Respiratory syncytial virus type B		2
Rhinovirus/enterovirus	8	9
*Bordetella pertussis*	1	1
*Mycoplasma pneumoniae*	1	1

**Table 3:** Discrepant results of LDT compared to the ePlex® RP panel

	LDT	ePlex® RP panel
Throat swab	Negative (retesting negative)	RV/EV
Nasopharyngeal swab	InfA (Cq 26) (enterovirus negative)	InfA-RV/EV
Nasal lavage	RV (Cq 39.1)	Negative
Sputum	MPV (Cq 30.3)	Negative (retesting MPV positive)
Sputum	InfA (Cq 33.1)	Negative (retesting negative)

*RV* rhinovirus; *EV* enterovirus; *InfA* influenza A; *MPV* metapneumovirus; *Cq* quantification cycle

In three patients, different sample types were tested (Table 4). From the first, a sputum and a throat swab were collected, of which only the first tested LDT positive for influenza A. The second tested rhinovirus positive in a nasal lavage, with LDT only, and negative in sputum. Of the third patient, a sputum and a throat swab were tested, of which only the sputum tested coronavirus 229E positive.

**Table 4:** Different sample types tested

Patient	Material	LDT	ePlex® RP panel
1	Sputum	InfA (Cq 33.1)	Negative
	Throat swab	Negative	Negative
2	Sputum	Negative	Negative
	Nasal lavage	RV (Cq 39.1)	Negative
3	Sputum	CoV 229E (Cq 33.4)	CoV 229E
	Throat swab	Negative	Negative

*RV* rhinovirus; *InfA* influenza A; *CoV* coronavirus; *Cq* quantification cycle

### Primary outcome measure: difference in time to result

For 62 of the 64 samples, both the time of acceptance and the time of result was recorded. The calculated time to result was significantly shorter, approximately 24 h, for the ePlex® RP panel than for LDT (*p* < 0.001) (Table 5). A time to result of over 35 h was seen with LDT in 15 samples, of which 13 had arrived on Friday and were tested on Monday. In the two remaining samples, there was a delay in requesting and authorization of the test subsequently. In the ePlex® RP panel, four samples had a time to result of more than 18 h. Two of these samples were already at the laboratory for several hours before the ePlex® RP panel testing was requested, while the testing of two samples was requested after 3:00 P.M. and, therefore, performed the next day (one due to failure of the initial sample).

**Table 5:** Time to result in hours of LDT compared to the ePlex® RP panel

Time to result	LDT	ePlex® RP panel	*p*-Value*
Median (h)	27.11	3.35	< 0.001
Range (h)	6.52–96.57	1.45–23.56	

*p-Value calculated with the Wilcoxon signed-rank test

### Secondary outcome measures: consequences for patient isolation

Of the 61 patients included in the analysis, 60 were admitted to the hospital at the time respiratory testing was requested. Fifty-one of these hospitalized patients were isolated while awaiting test results, whereas nine patients were not admitted in isolation. In these cases, isolation was not installed mainly because of low clinical suspicion of a pathogen requiring isolation. One of these nine patients needed isolation, since the ePlex® RP panel tested positive for influenza A (3 days ahead of LDT).

The tests showed that 19 out of 51 patients admitted in isolation had a respiratory pathogen requiring isolation. Of the remaining 32 patients, one died before test results became available and, for one patient, the duration of isolation was unknown, leaving 30 patients for further analysis. In 14 of these, isolation was discontinued based on the ePlex® RP panel results ahead of the LDT results. This resulted in a total reduction of 21 isolation days, with a median reduction of 2 days (range 1–4 days) per patient. In eight of the remaining patients, isolation was discontinued when the LDT results became available. In the other eight patients, of which three were children, isolation was not withdrawn at the moment LDT results were reported.

### Theoretical consequences for antiviral and antibacterial treatment

A total of 50 out of the 61 patients received antiviral or antibacterial treatment during hospitalization. Oseltamivir treatment was initiated in 19 patients awaiting test results, of which five tested positive for influenza A. In the 14 influenza ePlex® RP panel negative patients, oseltamivir could have been stopped approximately one day earlier (median of 22.59 h, range 5.33–72.03) based on the ePlex® RP results compared to LDT (Table 6). Of the total of 11 patients who tested influenza positive, the remaining six did not receive oseltamivir at the time of diagnosis. In one patient, oseltamivir treatment was started as soon as the ePlex® RP panel showed influenza A, one day prior to the LDT results, and one patient started when LDT was positive. Four patients did not receive any antiviral treatment, of which two were already dismissed at the time of definite LDT diagnosis.

**Table 6:** Theoretical median time in hours of isolation and treatment calculated based on the time to results

	No.	LDT (range)	ePlex® RP panel (range)	Difference (range)
Oseltamivir, h	14	27.08 (10.10–75.15)	3.38 (2.00–23.56)	22.59 (5.33–72.03)
Antibiotics atypical pneumonias, h	19	27.12 (8.27–81.11)	3.38 (1.52–23.56)	23.35 (−0.43–75.28)

Awaiting test results, 19 patients received antibiotic treatment for bacteria causing atypical pneumonias. In none of these patients, either the ePlex® RP panel or LDT (eight were tested) was positive for *Bordetella pertussis*, *Legionella pneumophila*, and *Mycoplasma pneumoniae*. In theory, in these 19 patients, a median duration of 23.35 h (range 0.43–75.28) antibiotic treatment for atypical pneumonia could have been saved, if treatment was stopped when the ePlex® RP panel tested negative.

### Additional diagnoses

Of the 61 patients, two tested positive by the ePlex® RP panel for a bacterial agent, one *Bordetella pertussis* and one *Mycoplasma pneumoniae*. In both patients, testing for these pathogens was not requested by the clinician and, as a consequence, not included in the routine diagnostic LDT workflow. The positive ePlex® RP panel results were confirmed by LDT with quantification cycle (Cq) values of 25.6 and 34.6 for *B. pertussis* and *M. pneumoniae*, respectively. LDT for atypical bacterial pathogens (*Mycoplasma pneumoniae*, *Chlamydia pneumoniae*, *Chlamydia psittaci*) was requested in only 16 patients. Legionella LDT was requested in only ten patients.

## Discussion

As hypothesized, diagnosis with the ePlex® RP assay significantly reduced the time to result (median 23.34 h) as compared to batch-wise LDT. Consequently, a total of 21 isolation days were saved and three days of influenza A exposure prevented. Unnecessary oseltamivir treatment could have been shortened at least 20 h in 14 patients and antibiotic treatment for atypical pneumonias by a median of 23.35 h in 19 patients. Proper therapeutic and isolation measurements could be installed in two patients for bacterial pathogens based on ePlex® RP panel detection that were not considered by the treating physicians and, therefore, not analyzed by routine LDT.

To our knowledge, this study is the first to report the use of the ePlex® RP panel in a clinical setting. It demonstrated a significant time reduction, reflecting previous clinical studies implementing rapid molecular testing [12–15], and significantly reduced the number of isolation days. Furthermore, confirmation of a single viral cause of infection in a cohort of patients enabled cohort nursing, which increased the number of isolation rooms available to patients awaiting identification of their respiratory pathogen. Efficient use of isolation rooms is essential during the influenza season, when the demand for these rooms is high.

The rapid ePlex® RP panel results could have resulted in a reduction of oseltamivir usage, which is in line with previous studies [14]. Results regarding reduction in antibiotic treatment for atypical pneumonias should be interpreted with care, while they are, according to the Dutch guidelines, only indicated for *Legionella pneumophila* in high-risk populations and can also been stopped based on negative urine antigen testing. The lack of routine testing for atypical respiratory bacterial pathogens (mostly *Legionella pneumophila*) and the finding of additional respiratory pathogens initially not considered by the clinicians underline the importance of syndromic respiratory testing.

Our study has a number of limitations. First, the clinical impact of our pilot study was hampered by its design. Since the ePlex® RP assay was readily offered to reduce the quest for isolation rooms during the coinciding influenza and respiratory syncytial virus epidemics early in 2017, its test results were not yet shown in the hospital information system but reported by phone, creating a bias. Moreover, the ePlex® RP panel result was reported as a provisional result awaiting routine LDT confirmation. The delay in showing the test results in the electronic patient record might have withheld clinicians to discontinue isolation and, therefore, created an underestimation of the true clinical potential. Furthermore, the findings of this study cannot be extrapolated readily, since this was a single-center study during just a part of one winter season. The benefits of rapid diagnostics might be more pronounced when assessing complete respiratory seasons.

So far, the ePlex® RP panel has been CE-IVD cleared for nasopharyngeal swabs only. However, especially samples from the lower respiratory tract such as sputum and bronchoalveolar lavage can be important to include in the CE-IVD clearance, since our study shows that these samples might have a higher diagnostic yield. However, in both our previous and current study, several different sample types were tested, with good results [7]. Nevertheless, the ePlex® RP panel had a failure rate of nearly 10%, in two cases due to internal control failure, while none of the LDT failed. Overall, the ePlex® RP panel results showed excellent concordance with our LDT; only three LDT positives (all with Cq values > 30) could not be detected using the ePlex® RP panel. This is in line with our previous findings reported by Nijhuis et al. [7]. The ePlex® RP panel is based on syndromic testing and has a standard panel containing the most common respiratory pathogens that are requested by the physician. However, the ePlex® RP panel is not complete, especially when caring for immunocompromised patients. In that case, additional LDT testing for *Legionella* species, cytomegalovirus, herpesvirus, toxoplasmosis, and fungal pathogens would still be necessary. Compared to LDT, ePlex® RP panel testing is more expensive regarding reagents and consumables, but cheaper with respect to hands-on time. In addition, rapid diagnostics will result in a cost reduction in the clinical departments, as demonstrated previously [16].

In conclusion, diagnosis of respiratory infections with the ePlex® RP assay resulted in a significant reduction in the time to result compared to LDT, which causes a reduction in isolation days and theoretically improved treatment regimens. Because of these advantages, we assume that this rapid diagnostic molecular assay will be of added value for ongoing improvement in patient care.

## References

[1] Bates M, Mudenda V, Mwaba P, Zumla A (2013). Deaths due to respiratory tract infections in Africa: a review of autopsy studies. Curr Opin Pulm Med.

[2] Lozano R, Naghavi M, Foreman K, Lim S, Shibuya K, Aboyans V, Abraham J, Adair T, Aggarwal R, Ahn SY, AlMazroa MA, Alvarado M, Anderson HR, Anderson LM, Andrews KG, Atkinson C, Baddour LM, Barker-Collo S, Bartels DH, Bell ML, Benjamin EJ, Bennett D, Bhalla K, Bikbov B, Bin Abdulhak A, Birbeck G, Blyth F, Bolliger I, Boufous S, Bucello C, Burch M, Burney P, Carapetis J, Chen H, Chou D, Chugh SS, Coffeng LE, Colan SD, Colquhoun S, Colson KE, Condon J, Connor MD, Cooper LT, Corriere M, Cortinovis M, de Vaccaro KC, Couser W, Cowie BC, Criqui MH, Cross M, Dabhadkar KC, Dahodwala N, De Leo D, Degenhardt L, Delossantos A, Denenberg J, Des Jarlais DC, Dharmaratne SD, Dorsey ER, Driscoll T, Duber H, Ebel B, Erwin PJ, Espindola P, Ezzati M, Feigin V, Flaxman AD, Forouzanfar MH, Fowkes FG, Franklin R, Fransen M, Freeman MK, Gabriel SE, Gakidou E, Gaspari F, Gillum RF, Gonzalez-Medina D, Halasa YA, Haring D, Harrison JE, Havmoeller R, Hay RJ, Hoen B, Hotez PJ, Hoy D, Jacobsen KH, James SL, Jasrasaria R, Jayaraman S, Johns N, Karthikeyan G, Kassebaum N, Keren A, Khoo JP, Knowlton LM, Kobusingye O, Koranteng A, Krishnamurthi R, Lipnick M, Lipshultz SE, Ohno SL, Mabweijano J, MacIntyre MF, Mallinger L, March L, Marks GB, Marks R, Matsumori A, Matzopoulos R, Mayosi BM, McAnulty JH, McDermott MM, McGrath J, Memish ZA, Mensah GA, Merriman TR, Michaud C, Miller M, Miller TR, Mock C, Mocumbi AO, Mokdad AA, Moran A, Mulholland K, Nair MN, Naldi L, Narayan KM, Nasseri K, Norman P, O’Donnell M, Omer SB, Ortblad K, Osborne R, Ozgediz D, Pahari B, Pandian JD, Rivero AP, Padilla RP, Perez-Ruiz F, Perico N, Phillips D, Pierce K, Pope CA, Porrini E, Pourmalek F, Raju M, Ranganathan D, Rehm JT, Rein DB, Remuzzi G, Rivara FP, Roberts T, De León FR, Rosenfeld LC, Rushton L, Sacco RL, Salomon JA, Sampson U, Sanman E, Schwebel DC, Segui-Gomez M, Shepard DS, Singh D, Singleton J, Sliwa K, Smith E, Steer A, Taylor JA, Thomas B, Tleyjeh IM, Towbin JA, Truelsen T, Undurraga EA, Venketasubramanian N, Vijayakumar L, Vos T, Wagner GR, Wang M, Wang W, Watt K, Weinstock MA, Weintraub R, Wilkinson JD, Woolf AD, Wulf S, Yeh PH, Yip P, Zabetian A, Zheng ZJ, Lopez AD, Murray CJ (2012). Global and regional mortality from 235 causes of death for 20 age groups in 1990 and 2010: a systematic analysis for the Global Burden of Disease Study 2010. Lancet.

[3] Lim SS, Vos T, Flaxman AD, Danaei G, Shibuya K, Adair-Rohani H, AlMazroa MA, Amann M, Anderson HR, Andrews KG, Aryee M, Atkinson C, Bacchus LJ, Bahalim AN, Balakrishnan K, Balmes J, Barker-Collo S, Baxter A, Bell ML, Blore JD, Blyth F, Bonner C, Borges G, Bourne R, Boussinesq M, Brauer M, Brooks P, Bruce NG, Brunekreef B, Bryan-Hancock C, Bucello C, Buchbinder R, Bull F, Burnett RT, Byers TE, Calabria B, Carapetis J, Carnahan E, Chafe Z, Charlson F, Chen H, Chen JS, Cheng AT, Child JC, Cohen A, Colson KE, Cowie BC, Darby S, Darling S, Davis A, Degenhardt L, Dentener F, Des Jarlais DC, Devries K, Dherani M, Ding EL, Dorsey ER, Driscoll T, Edmond K, Ali SE, Engell RE, Erwin PJ, Fahimi S, Falder G, Farzadfar F, Ferrari A, Finucane MM, Flaxman S, Fowkes FG, Freedman G, Freeman MK, Gakidou E, Ghosh S, Giovannucci E, Gmel G, Graham K, Grainger R, Grant B, Gunnell D, Gutierrez HR, Hall W, Hoek HW, Hogan A, Hosgood HD, Hoy D, Hu H, Hubbell BJ, Hutchings SJ, Ibeanusi SE, Jacklyn GL, Jasrasaria R, Jonas JB, Kan H, Kanis JA, Kassebaum N, Kawakami N, Khang YH, Khatibzadeh S, Khoo JP, Kok C, Laden F, Lalloo R, Lan Q, Lathlean T, Leasher JL, Leigh J, Li Y, Lin JK, Lipshultz SE, London S, Lozano R, Lu Y, Mak J, Malekzadeh R, Mallinger L, Marcenes W, March L, Marks R, Martin R, McGale P, McGrath J, Mehta S, Memish ZA, Mensah GA, Merriman TR, Micha R, Michaud C, Mishra V, Mohd Hanafiah K, Mokdad AA, Morawska L, Mozaffarian D, Murphy T, Naghavi M, Neal B, Nelson PK, Nolla JM, Norman R, Olives C, Omer SB, Orchard J, Osborne R, Ostro B, Page A, Pandey KD, Parry CD, Passmore E, Patra J, Pearce N, Pelizzari PM, Petzold M, Phillips MR, Pope D, Pope CA, Powles J, Rao M, Razavi H, Rehfuess EA, Rehm JT, Ritz B, Rivara FP, Roberts T, Robinson C, Rodriguez-Portales JA, Romieu I, Room R, Rosenfeld LC, Roy A, Rushton L, Salomon JA, Sampson U, Sanchez-Riera L, Sanman E, Sapkota A, Seedat S, Shi P, Shield K, Shivakoti R, Singh GM, Sleet DA, Smith E, Smith KR, Stapelberg NJ, Steenland K, Stöckl H, Stovner LJ, Straif K, Straney L, Thurston GD, Tran JH, Van Dingenen R, van Donkelaar A, Veerman JL, Vijayakumar L, Weintraub R, Weissman MM, White RA, Whiteford H, Wiersma ST, Wilkinson JD, Williams HC, Williams W, Wilson N, Woolf AD, Yip P, Zielinski JM, Lopez AD, Murray CJ, Ezzati M (2012). A comparative risk assessment of burden of disease and injury attributable to 67 risk factors and risk factor clusters in 21 regions, 1990–2010: a systematic analysis for the Global Burden of Disease Study 2010. Lancet.

[4] Pfuntner A, Wier LM, Stocks C (2013) Most frequent conditions in U.S. hospitals. Accessed 17 Mar 2017

[5] Fine MJ, Auble TE, Yealy DM, Hanusa BH, Weissfeld LA, Singer DE, Coley CM, Marrie TJ, Kapoor WN (1997). A prediction rule to identify low-risk patients with community-acquired pneumonia. N Engl J Med.

[6] GenMark. Respiratory Pathogen (RP) Panel. https://www.genmarkdx.com/int/solutions/panels/eplex-panels/respiratory-pathogen-panel/. Accessed 7 Jun 2017

[7] Nijhuis RH, Guerendiain D, Claas EC, Templeton KE (2017). Comparison of ePlex respiratory pathogen panel with laboratory-developed real-time PCR assays for detection of respiratory pathogens. J Clin Microbiol.

[8] Loens K, van Loon AM, Coenjaerts F, van Aarle Y, Goossens H, Wallace P, Claas EJ, Ieven M (2012). Performance of different mono- and multiplex nucleic acid amplification tests on a multipathogen external quality assessment panel. J Clin Microbiol.

[9] Templeton KE, Scheltinga SA, van der Zee A, Diederen BM, Kruijssen AM, Goossens H, Kuijper E, Claas EC (2003). Evaluation of real-time PCR for detection of and discrimination between Bordetella pertussis, Bordetella parapertussis, and Bordetella holmesii for clinical diagnosis. J Clin Microbiol.

[10] Templeton KE, Scheltinga SA, Sillekens P, Crielaard JW, van Dam AP, Goossens H, Claas ECJ (2003). Development and clinical evaluation of an internally controlled, single-tube multiplex real-time PCR assay for detection of Legionella pneumophila and other Legionella species. J Clin Microbiol.

[11] Op den Buijs IOM, Roymans RT, van de Bovenkamp JHB (2014) Development of a multiplex qPCR for the detection of atypical pneumonia using the automated MAX™ system. NVMM Spring Meeting

[12] Brendish NJ, Malachira AK, Armstrong L, Houghton R, Aitken S, Nyimbili E, Ewings S, Lillie PJ, Clark TW (2017). Routine molecular point-of-care testing for respiratory viruses in adults presenting to hospital with acute respiratory illness (ResPOC): a pragmatic, open-label, randomised controlled trial. Lancet Respir Med.

[13] Muller MP, Junaid S, Matukas LM (2016). Reduction in total patient isolation days with a change in influenza testing methodology. Am J Infect Control.

[14] Chu HY, Englund JA, Huang D, Scott E, Chan JD, Jain R, Pottinger PS, Lynch JB, Dellit TH, Jerome KR, Kuypers J (2015). Impact of rapid influenza PCR testing on hospitalization and antiviral use: a retrospective cohort study. J Med Virol.

[15] Rogers BB, Shankar P, Jerris RC, Kotzbauer D, Anderson EJ, Watson JR, O’Brien LA, Uwindatwa F, McNamara K, Bost JE (2015). Impact of a rapid respiratory panel test on patient outcomes. Arch Pathol Lab Med.

[16] Poelman R, van der Meer J, van Leer-Buter C, Riezebos-Brilman A, Niesters HGM (2015). Point-of-impact testing in the emergency department: rapid diagnostics for respiratory viral infections. J Clin Virol.

